# Tobacco Use Patterns

**DOI:** 10.1155/2012/564390

**Published:** 2012-12-26

**Authors:** Timothy A. McAfee, Judy Kruger

**Affiliations:** ^1^Office on Smoking and Health, NCCDPHP, CDC, 4770 Buford Highway, MS-K50, Atlanta, GA 30341, USA; ^2^Epidemiology Branch, Office on Smoking and Health, NCCDPHP, CDC, 4770 Buford Highway, MS-K50, Atlanta, GA 30341, USA

The use of combustible tobacco products (e.g., cigarettes, cigars, pipe, bidis, kreteks, and hookah) among adults remains widespread around the world. Unless dramatic progress is made diminishing the initiation and increasing cessation of combustible tobacco product use, a billion preventable deaths will occur in the 21st century [1]. These deaths will be accompanied by unimaginable human suffering and unaffordable economic loss from both preventable healthcare expenditures and loss of productivity from early death and disease. Health risks not only impact the smoker, but also hundreds of millions of individuals who inhale secondhand smoke from combustible tobacco products. In addition to the risks associated with smoking tobacco products, there are important concerns associated with the use of noncombustible tobacco products (e.g., chew, dip, snus, Gutka, and Ikmik). Concerns include both direct health effects from high-toxicant products, particularly in Southern Asia that account for the majority of global noncombustible use and the impact of dual use of noncombustible products with smoked tobacco products [2, 3]. This is a pattern being seen in youth, young adults [4, 5], and adults in the United States (as in the paper of R. McMillen et al. “*Use of emerging tobacco products in the United States*”), potentially increasing initiation and prolonging smoking. 

In order to successfully tackle the immense challenges ahead, it is critical that public health workers and others committed to eradicating the harm caused by the tobacco epidemic have a full understanding of what is actually happening. Questions need to be answered such as: How are tobacco use patterns evolving? How are changes in the design of tobacco products impacting health outcomes? How are emerging tobacco products being marketed? and, How is their use impacting the use of combustible tobacco products? Which tobacco control policies and interventions work for different populations and use patterns? Few health risks have received as much attention from researchers and policy makers as the use of tobacco products. Articles recently published in the Journal of Environmental and Public Health can inform health policy decisions in several ways: by providing information on specific populations who use these products, by targeting interventions to products and users, and by identifying and characterizing emerging products developed and marketed by the tobacco industry. Scientific research, surveillance, and evaluation are valuable tools for informing health policy decisions because they can identify the introduction of new products, offer insight as to the prevalence of use of those products, and provide information on the effectiveness of specific interventions and tobacco control policy. 

In the United States, 15.8% of high school students [6] and 19.3% of adults smoke cigarettes [7]. Attention to the marketing of new tobacco products and combinations of use of these products is essential to ending the tobacco epidemic. Current tobacco use trend indicators may provide insufficient information as the tobacco industry innovates its products and strategies. For example, a primary indicator of progress in the tobacco epidemic has been the prevalence of cigarette smoking and cigarette consumption. However, because of differences in taxation between cigarettes and other combustible products and lack of FDA authority to regulate flavoring and other product characteristics of cigars and pipe tobacco, consumption and prevalence of use of cigars and pipe tobacco (used in roll-your-own cigarettes) is increasing dramatically [8, 9]. An over-estimation of progress in tobacco control, particularly among youth and young adults, would result if we do not pay appropriate attention to increases in use of these other tobacco products. 

Another example highlighted in this issue in the paper by R. Sacks et al. “Exploring the next frontier for tobacco control: nondaily smoking among New York city adults” is a trend towards decreased consumption of cigarettes among smokers, resulting from both an increase in nondaily smoking and a decrease in the average number of cigarettes smoked per day. How is this trend evolving? What population groups are most affected? Have risk perceptions altered, including the perception of being a smoker? Conversely, there is concern that the percent of the smoking population with significant mental health or substance abuse disorders is increasing [10], but this information is less routinely collected in surveys. What are the implications of these trends for clinical interventions, media and education campaigns, and public policy approaches that were designed and tested in a time when most people who smoked did so daily, smoked a pack of cigarettes or more a day, and where people who had mental health and substance abuse disorders were often excluded from studies? 

Federal and state tobacco control efforts will benefit greatly from the information provided by studies such as those found within this special issue, especially given the new potential of regulatory action to profoundly diminish the death, disease, and suffering caused by tobacco use. With the authority to regulate toxicant exposure, including potentially requiring the lowering of nicotine content to nonaddictive levels, major alterations in the tobacco product marketplace are possible, especially if combined with proven nonregulatory tobacco control interventions such as raising the price of tobacco products, eliminating secondhand smoke exposure, fully funding tobacco control programs (including high-impact tobacco control mass media campaigns), and barrier-free access to quit help for those who want it. However, while this new path presents opportunity, it is also fraught with challenges and even peril. The role of broad-spectrum research ranging from surveillance, to laboratory studies, to market research is essential to help evaluate the impact, as well as monitor for unanticipated consequences of these interventions, which will help guide policy evolution and provide needed support for the regulatory process. 

The goal of this special issue is to educate the readership of the Journal of Environmental and Public Health about emerging tobacco products and their patterns of use and to stimulate research in tobacco control. Through these and other papers, we hope to provide information that will eventually have an impact on reducing the number of tobacco users and help those who use it to quit. The list of topics is broad and impressive; the studies cover a wide range of areas: prevalence; secondhand smoke exposure; dual use; smoking cessation efforts; and product design.
